# Barriers to the access of people with disabilities to health services: a scoping review

**DOI:** 10.11606/s1518-8787.2022056003893

**Published:** 2022-06-27

**Authors:** Karina Aparecida Padilha Clemente, Simone Vieira da Silva, Gislene Inoue Vieira, Maritsa Carla de Bortoli, Tereza Setsuko Toma, Vinícius Delgado Ramos, Christina May Moran de Brito

**Affiliations:** I Universidade de São Paulo Faculdade de Medicina Departamento de Medicina Legal, Ética Médica, Medicina Social e do Trabalho São Paulo SP Brasil Universidade de São Paulo. Faculdade de Medicina. Departamento de Medicina Legal, Ética Médica, Medicina Social e do Trabalho. São Paulo, SP, Brasil.; II Secretaria de Estado da Saúde de São Paulo Instituto de Saúde Departamento de Ciência e Tecnologia São Paulo SP Brasil Secretaria de Estado da Saúde de São Paulo. Instituto de Saúde. Departamento de Ciência e Tecnologia. São Paulo, SP, Brasil.; III Hospital das Clínicas Faculdade de Medicina Universidade de São Paulo São Paulo SP Brasil Hospital das Clínicas da Faculdade de Medicina da Universidade de São Paulo. Instituto de Medicina Física e Reabilitação. São Paulo, SP, Brasil.; IV Hospital das Clínicas Faculdade de Medicina Universidade de São Paulo São Paulo SP Brasil Hospital das Clínicas da Faculdade de Medicina da Universidade de São Paulo. Instituto do Câncer do Estado de São Paulo. São Paulo, SP, Brasil.

**Keywords:** Disabled Persons, Barriers to Access of Health Services, Communication Barriers, Physician-Patient Relations, Health Services for Persons with Disabilities, Review

## Abstract

**OBJECTIVE:**

To analyze the scientific evidence regarding barriers to the access of people with disabilities to health services.

**METHODS:**

A scoping review was carried out from the main question: “What are the main barriers that people with disabilities face in accessing health services?” The articles were surveyed in July 2019 in six scientific literature databases. Of the 1,155 documents identified in the searches, after selection by title and abstract, 170 publications were read in full and, thus, 96 articles were included and categorized according to the theoretical framework.

**RESULTS:**

The main barriers indicated by the users of the service were: communication failure between professionals and patient/caregiver; financial limitations; attitudinal/behavioral issues; scarce service provision; organizational and transport barriers. The main barriers presented by service providers were: lack of training to professionals; failure of the health system; physical barriers; lack of resources/technology; and language barriers.

**CONCLUSIONS:**

It was evident that people with disabilities face several barriers when trying to access the health services they need and that users and health professionals have distinct and complementary views on difficulties.

## INTRODUCTION

The *Lei Brasileira de Inclusão da Pessoa com Deficiência* (LBI – Brazilian Law for the Inclusion of Persons with Disabilities) incorporates into the Brazilian legal system the current concept of disability that considers it the result of the interaction between people with physical, mental, intelectual, and sensory impairments and disabling environmental barriers^1^.

According to the World Health Organization (WHO) there are approximately one billion people with disabilities, making up 15% of the world’s population^2^. In Brazil, the 2010 Demographic Census estimated that 23.9% of people had some type of self-reported disability and that 6.7% of them considered them “severe” disabilities^3^. With the aging of the population and the increasing prevalence of chronic non-communicable and disabling diseases, and of harmful behaviors that can affect health, these numbers are likely to increase^2^.

These people may have specific health needs due to their disabilities and associated health conditions. Evidence indicates that people with disabilities have greater health needs than people without disabilities and that these needs are generally not met. In addition to the common health needs, such as immunization, they have worse levels of access to health services and worse health outcomes, especially in low, medium/low, and medium/high income countries, such as Brazil^2^. This situation is indeed evident in our country, where studies indicate that people with disabilities show higher levels of morbidity, behaviors that have a negative impact on health, and greater use of health services and hospitalizations^4^.

People with disabilities, therefore, need adequate general health care that covers all aspects, including prevention and health promotion, based on primary, secondary, and tertiary care, and may also require rehabilitation care and specialized treatment – related or not to their underlying disability – to “optimize functionality and reduce disability”^2^.

There is strong evidence around the world that people with disabilities have difficulties in accessing health services and the scarce data available about research conducted in Brazil also show this. For example, the Brazilian National Health Survey showed a low level of access to rehabilitation services among this population (5–30% in all types of disability), with some geographical variation^5^. Although Brazilian policies and laws strongly support the inclusion of this group in the health system, the Zika epidemic in 2015 revealed gaps in the Unified Health System (SUS) in terms of health care for people with disabilities. Caregivers of children with congenital Zika virus syndrome have great difficulties in accessing relevant services, centered on people, both locally and nationally^6^.

Although fundamental for the performance of health systems worldwide, access to health care by people with and without disabilities remains a complex concept^7^. Potential challenges faced in accessing health care include discrimination, physical inaccessibility, information inaccessibility and unavailability. Around the world, evidence on how to promote the inclusion of people with disabilities in the health system is lacking^2^.

Based on the problem exposed, we carried out a scoping review, whose objective was to analyze global and local scientific evidence on barriers for the access of people with disabilities to health services.

## METHODS

According to the Joanna Briggs Institute Reviewer’s Manual^8^, a scoping review consists of the following phases: 1) identify the research question; 2) identify relevant studies; 3) select the available studies; 4) map the data; 5) collect, summarize, and report the results; and 6) perform the consultation exercise, which is optional and was not applied in this study. This type of knowledge synthesis has an original structure and must be conducted with rigor, transparency, and reliability. It has been used by researchers interested in mapping the scientific literature and detecting knowledge gaps^8^. This manuscript was anchored by a specific version of PRISMA (Preferred Reporting Items for Systematic reviews and Meta-Analyses extension for Scoping Reviews, PRISMA, 2020), a 22-item checklist designed to guide the development of a scoping review report^9^.

The proposed guide question for this review was: “What are the main barriers that people with disabilities face in accessing health services?”

This study, to explore the findings from a comprehensive and dynamic perspective, adopts the proposal of Levesque et al.^7^, who structure the concept of access to health care at multiple levels and identify the determinants that can affect it from factors related to health systems, institutions, and professionals, and individuals, communities, and the population.

A systematic search for evidence published in Portuguese, English, or Spanish, was conducted addressing the themes: people with disabilities, accessibility to health services, health services for people with disabilities, availability of health services, disparities and inequities in health, health care accessibility, health services accessibility. The descriptors were accompanied by the Boolean operators “*or*” and “*and*” in the searches.

The articles were surveyed in July 2019 in six scientific literature databases: PubMed, Health Systems Evidence, Scopus, EMBASE, Health Evidence, and Lilacs as described in the Box.

**Box:** Detailing of the bibliographic survey carried out in scientific literature databases.

Base	Date	Strategy
PubMed	07/06/2019	(((Disabled Person OR Person, Disabled OR Persons, Disabled OR Persons with Disabilities OR Disabilities, Persons with OR Disability, Persons with OR Persons with Disability OR Handicapped OR People with Disabilities OR Disabilities, People with OR People with Disability OR Physically Handicapped OR Handicapped, Physically OR Physically Disabled OR Disabled, Physically OR Physically Challenged)) AND (“Health Services Accessibility”[Mesh] OR Availability of Health Services OR Health Services Availability OR Accessibility, Health Services OR Access to Health Care OR Accessibility of Health Services OR Health Services Geographic Accessibility OR Program Accessibility OR Accessibility, Program)) AND (SYSTEMATIC REVIEW OR SYSTEMATIC REVIEWS)
PubMed	07/06/2019	(((Disabled Person OR Person, Disabled OR Persons, Disabled OR Persons with Disabilities OR Disabilities, Persons with OR Disability, Persons with OR Persons with Disability OR Handicapped OR People with Disabilities OR Disabilities, People with OR People with Disability OR Physically Handicapped OR Handicapped, Physically OR Physically Disabled OR Disabled, Physically OR Physically Challenged)) AND ((“Health Services for Persons with Disabilities”[Mesh] OR Health Services for the Disabled OR Health Services for People with Disabilities OR Health Services for Disabled Persons) AND Review[ptyp])) AND (SYSTEMATIC REVIEW OR SYSTEMATIC REVIEWS)
PubMed	07/06/2019	(((Disabled Person OR Person, Disabled OR Persons, Disabled OR Persons with Disabilities OR Disabilities, Persons with OR Disability, Persons with OR Persons with Disability OR Handicapped OR People with Disabilities OR Disabilities, People with OR People with Disability OR Physically Handicapped OR Handicapped, Physically OR Physically Disabled OR Disabled, Physically OR Physically Challenged)) AND ((“Healthcare Disparities”[Mesh] OR Disparity, Healthcare OR Health Care Inequalities OR Health Care Inequality OR Inequalities, Health Care OR Inequality, Health Care OR Healthcare Disparity OR Healthcare Inequalities OR Healthcare Inequality OR Inequalities, Healthcare OR Inequality, Healthcare OR Disparities, Healthcare OR Health Care Disparities OR Disparities, Health Care OR Disparity, Health Care OR Health Care Disparity) AND Review[ptyp])) AND (SYSTEMATIC REVIEW OR SYSTEMATIC REVIEWS)
Health Systems Evidence	07/06/2019	disability OR disabilities
Health Evidence	07/06/2019	[(disabled persons) OR disability OR disabilities]
Scopus	07/06/2019	TITLE-ABS-KEY(“disabled persons”ANDaccessibility)AND(LIMIT-TO(DOCTYPE,”re”))
EMBASE	07/06/2019	(‘disabled person’/exp OR ‘disabled person’ OR ‘disability’/exp OR disability) AND (‘accessibility’/exp OR accessibility) AND (‘health care facilities and services’/exp OR ‘health care facilities and services’) AND [review]/lim AND [embase]/lim
Lilacs via VHL regional portal	07/06/2019	(“pessoas com deficiência” OR “disabled persons” OR “personas con discapacidad” OR deficiência OR deficiências OR “deficiência física” OR “deficiências fisicas” OR “deficiente físico” OR incapacidade OR “incapacidade funcional” OR “limitação física” OR “pessoa com desvantagem” OR “pessoas com desvantagens” OR “pessoa com incapacidade” OR “pessoas com incapacidade” OR “pessoas com deficiências” OR “pessoas com incapacidades” OR “pessoa com incapacidade física” OR “pessoa com deficiência fisica” OR “pessoas com deficiência física” OR “pessoas com incapacidade física” OR “pessoas com deficiências físicas” OR “pessoa com limitação física” OR “pessoas com limitação física” OR “pessoas com limitações físicas” OR “pessoa com necessidade especial” OR “pessoas com necessidade especial” OR “pessoas com necessidades especiais”) AND (“acesso aos serviços de saúde” OR “health services accessibility” OR “accesibilidad a los servicios de salud” OR “disparidades em assistência à saúde” OR “healthcare disparities” OR “disparidades en atención de salud” OR “serviços de saúde para pessoas com deficiência” OR “health services for persons with disabilities” OR “servicios de salud para personas con discapacidad”) AND (instance:”regional”) AND ( db:(“LILACS” OR “BDENF” OR “BBO” OR “IBECS”))

The process of selecting articles by reading titles, abstracts, and the full text was carried out in subsequent stages by a pair of reviewers, independently. Disagreements were decided by a third reviewer.

The focus of this review was literature reviews addressing physical, hearing, visual, intellectual, and multiple disabilities, not covering mental health conditions.

Articles that addressed a specific health condition or need or compared different forms of treatment, which did not present questions related to the access of people with disabilities to the health system and publications that were not presented as a review of the literature were excluded.

The main information of the publications was summarized in a spreadsheet created specifically for this review, aiming to guide the descriptive and critical analyses of the selected studies. The extraction process was not performed in duplicate.

The extraction worksheet contained the following data: database from which the articles were collected; title; author; year of publication; bibliographic reference; study design; number and design of studies that were included in each publication; last year of search; objectives; primary and secondary foci; types of disability (visual, hearing, physical, intellectual, or multiple disabilities); study population and health condition of the individuals who composed the samples; context of the interventions analyzed; levels of health services covered (primary, secondary, or tertiary); countries where the studies were conducted; state or region in the case of national articles; information on the health systems contained in the articles; relevant knowledge gaps raised; and observations on the study.

Information on which components of the health system were related to the barriers were also extracted from the articles, with the theoretical reference of the publication “Strengthening Health Systems to Improve Health Outcomes – Everybody’s Business” from the World Health Organization (WHO)^10^, which considers the following areas: leadership and governance; workforce; financing; technologies; information systems; and service delivery.

Information on equity was collected, when available, according to the theoretical framework proposed by O’Neill et al.^11^, summarized in the acronym Progress (Place of Residence, Race, Occupation, Gender, Religion, Education, Socioeconomic status, and Social capital) plus factors that may lead to discrimination, factors related to caregivers and personal relationships.

The central extraction category comprised barriers and facilitators of access, identified from the perspectives of people with disabilities, caregivers, professionals, services, and health system.

The reviews included were not evaluated for methodological quality, since the stage is optional in a scoping review.

Etymologically, the term access presents complex notions. “Access is a dimension of the performance of the health system, associated with provision,” whereas accessibility refers to the “characteristic of the health service provision or the adjustment between provision and population,” which can be measured by analyzing the results of performance evaluation programs in the case of health services^12^.

Regarding the health context, Levesque et al.^7^ propose a dynamic structure composed of characteristics of health service providers and of skills acquired by users of these services. The dimensions of access are not independent, they are interrelated and may influence each other. In this context, access is conceived in five dimensions and as five skills.

The five characteristics manifested by systems, institutions, organizations, and health providers, as well as their particularities, are: approachability acceptability, availability and accommodation, affordability, and appropriateness.

The five skills assigned to individuals, families, communities, and populations are: ability to perceive, ability to seek, ability to reach, ability to pay, and ability to engage.

An articulated dynamic composed of practical actions that interact with each other is evident. Depending on the quality of this interaction, the dimensions of access and skills may constitute barriers or facilitators.

Furthermore, Federal Law 13.146/2015, LBI^1^, also known as the Statute of Persons with Disabilities, in its 3rd Article, specifically presents six types of barriers that can hinder or make it impossible for people with disabilities to access health services. The barriers are divided into: urbanistic, architectural, transportation, communication, attitudinal, and technological.

Thus, from the extracted data, we analyzed the barriers categorized in terms of the use of health services (by users and caregivers) and the provision of these services (by professionals, services and systems), which were grouped according to the five dimensions of access^7^ and the six possibilities of barriers presented in the LBI^1^.

## RESULTS

Of the 1,155 documents identified in the searches, we excluded 234 publications for being duplicates, totaling 921 articles for initial analysis. After selection by titles and abstracts, we searched 170 articles in full and then evaluated 158 articles for eligibility, of which 62 were excluded for non-relevance to the research objective. Thus, we included 96 articles and extracted and categorized their data. The entire selection and eligibility process is represented in the Figure.

**Figure:**
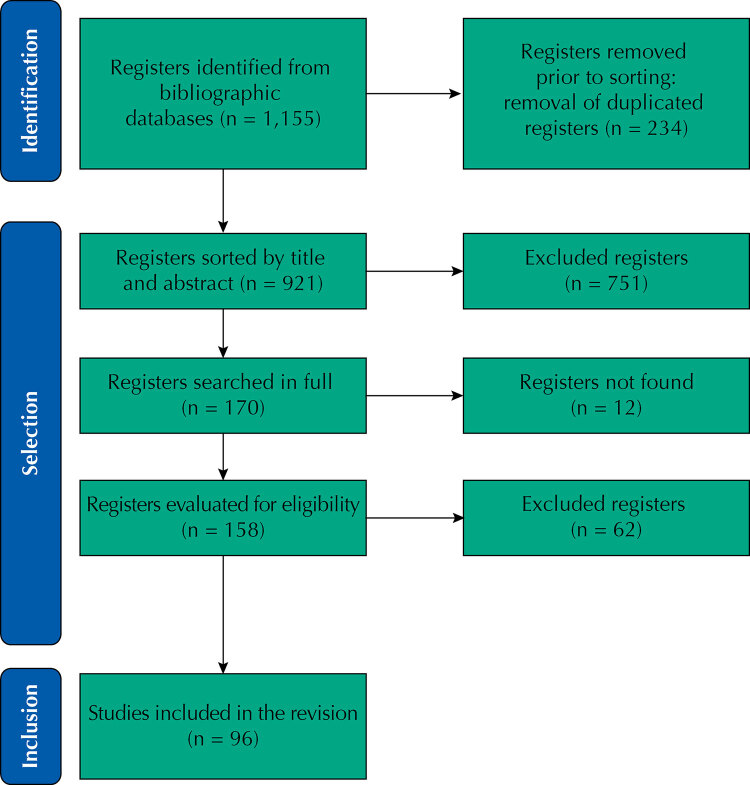
Studies’ selection flowchart, adapted from PRISMA^9^.

The countries of the studies included were classified according to criteria established by the WHO Standard Country or Area Code for Statistical Use, originally published as Series M, No. 49 and now commonly referred to as M49 standard^13^.

Most of the selected articles analyze data from countries in the American region (66.6%, n = 64), of which 90% (n = 58) are from North America. Following, European countries are the subject of 55.2% (n = 53) of publications, followed by Oceania (n = 36), and Asia (n = 33) with just over 30% each and, finally, Africa (6.2%, n = 6). Note that some articles include data from more than one country or region and that 36.4% (n = 35) do not specify where they were carried out.

Of the 96 publications included, only four highlight studies conducted in Brazil^14^. Regarding the types of disabilities found, 21% of the populations studied had physical disabilities; 17% intellectual; 8% visual; 7% hearing; 2% multiple disabilities; and 1% cerebral palsy. In 44% of the studies, the authors did not specify the types of disability of the studied population.

Regarding the level of complexity of health care covered by the studies, the contexts of intervention in which they are inserted varied. Whereas more than a third of articles leaves the level of care unspecified (n = 38), others involve more than one level or even all three levels. Of the total, 26 studies involve primary care, 30 studies involve specialized care, and 35 studies involve tertiary care.

Regarding the years in which the articles were published, the oldest was published in 1997 (n = 1) and the most recent are from 2019 (n = 2). The number of articles published remarkably increases over the years, with only four articles in 2008, nine in 2011, 12 in 2014, and 15 in 2017.

In the data analysis, we divided the barriers identified by users and caregivers from the barriers identified by health service providers.

In 10 reviews, the barriers presented by users, caregivers and health service providers, in general, are nonspecific in terms of level of care. In service provision, 11 reviews are non-specific in terms of level of care.

About the barriers found in the studies, Tables 1 and 2 show those related to the use or provision of services, according to the levels of care, demonstrating respectively the perception of users and service providers. Considering the use of services, eight reviews reported barriers related to primary care, four to specialized care, and two to tertiary level. Six reviews are dedicated to the barriers identified by service providers at primary care, five on specialized care, and three on tertiary level.

Table 1 shows the barriers to the access of people with disabilities to health services, according to the level of care and the perception of users.

**Table 1:** Barriers to access of people with disabilities in health services, according to the level of care (users).

Level of care	Barriers found in the literature
Basic	Limited access to psychological support and little or no community-based psychiatric support^18^; difficulty in finding physicians willing to care for severely disabled and complex patients^19^; difficulty in finding the needed health services^18^^,^^19^; lack of request for and performance of preventive tests and care^20^; lack of access to information on reproductive sexual health, family planning and prenatal and postnatal services^21^; lack of professional support^22^; lack of communication of the professional with the patient or caregiver^22–25^; lack of information to caregivers about the patient’s health conditions and insufficient access to child care services^25^; lack of health insurance and inability to pay for any service expenses^18^^,^^20^^,^^25^; and fear and anxiety regarding the treatment of children with disabilities^20^.
Specialized	Lack of access to health services^26^; lack of communication and access to information^27–29^; lack of involvement in making decisions about their health^28^; and problem of location of health units and transport difficulties to reach the services, as well as the cost of transportation^29^.
High complexity	Organizational, social, and physical barriers to access health services^30^; lack of information about their health and treatment^31^; and feeling of abandonment by caregivers regarding the treatment of users^31^.
Unspecified level of attention	Difficulties with transportation^32^^,^^33^; financial difficulties^14,16,34–36^; discrimination from health service providers^14,16,34–36^; lack of patient support in the rehabilitation process^32^; lack of communication by professionals^37^^,^^38^; lack of community awareness regarding disability^32^; lack of caregivers for adults with physical disabilities^39^; lack of “accessible” information on treatment and health services^34^^,^^35^^,^^37^^,^^38^; and a long waiting period for hospital visits after referral^38^.

Table 2 shows the barriers to the access of people with disabilities to health services, according to the level of care, from the perspective of service provision.

**Table 2:** Barriers to the access of people with disabilities in health services, according to the level of care (service provision).

Level of care	Barriers found in the literature
Basic	Lack of reliable and accessible transportation for users to arrive at consultations^20^^,^^22^^,^^25^; lack of training and inadequate skills on the part of professionals, environmental issues such as distant health units, insufficient time for consultations, negative attitude and lack of coordinated care by professionals^18^^,^^20^; and failures of health systems in responding adequately to the identified treatable morbidity^40^.
Specialized	Poor access to buildings, difficulty in transporting wheelchairs and poor parking facilities for disabled people, community and social environments generally unsuitable for wheelchair access^41^; difficulty for timely access to appropriate services^27^; lack of care for secondary health care^26^; lack of training and skills necessary for professionals and fragmentation of the health system^28^^,^^42^; and long waiting time for care and inadequate procedures for treatments and maintenance of assistive products^41^.
High complexity	Lack of self-care oriented to health policies and promotion, accessibility difficulties, limited availability of trained professionals, lack of social support, discrimination, financial restrictions, lack of information and educational interventions directed to users^43^; lack of communication by professionals^31^^,^^44^; and lack of funding for the purchase of devices necessary for treatment^44^.
Unspecified level of attention	Barrier to the use of technology by therapists, lack of training and/or resources to support the use of technology in the practice^33^; communication barriers between professionals and patients and among the health team itself^16^^,^^32^^,^^34^; language and cultural comprehension barriers^35^; physical barriers to care^16^; barriers to rehabilitation financing^45^ and technologies^33^; barriers to performing health exams^38^; discrimination from the health service provider^34^^,^^36^; failure to schedule appointments^32^; lack of skills of professionals^46^; lack of education initiatives on health services and misunderstanding of rehabilitation by people with disabilities^32^; lack of personal motivation of professionals^32^; lack of patience of professionals, lack of empathy, little understanding of disability issues^16^^,^^37^; lack of resources in the areas of psychology, speech therapy, and neuropsychology^32^; and fear and anxiety among professionals^36^.

Considering the components of the theoretical framework of Levesque et al.^7^ (2013) and LBI^1^, Table 3 shows the barriers categorized according to the list of “who receives” (users and caregivers).

**Table 3:** Barrier categories according to components of the theoretical framework from Levesque et al.^7^ (2013) and LBI^1^ – users and caregivers.

Use of the service: individuals, families, communities, and populations						
Barriers	Ability to perceive	Ability to seek	Ability to reach	Ability to pay	Ability to engage	Others
Urbanistic			Problem of location of health units^29^.			
Architectural			Organizational, social, and physical barriers to access health services^30^.			
Transport			Transport difficulties^29^^,^^32^^,^^33^ to reach health units, and the cost of transportation^29^.			
Communication			Lack of access to information about their health^27–29^, about treatment and health services ^31,34–37^, and about reproductive sexual health^21^; and lack of communication^37^ of the professional with the patient or caregiver^22^^,^^25^^,^^37^^,^^38^.			
Attitudinal	Lack of access to family planning services and prenatal and postnatal services^21^; insufficient access to care services for children^25^; lack of access to health services^28^; feeling of abandonment by caregivers regarding the treatment of users^31^; discrimination against health service providers^14,16,34–36^; and lack of patient support in the rehabilitation process^32^.	Fear and anxiety regarding the treatment of children with disabilities^20^; lack of professional support^22^; limited access to psychological support and little or no community-based psychiatric support^18^; difficulty in finding physicians willing to care for severely disabled and complex patients^19^; and difficulty in finding health services that they need^18^^,^^19^.				Lack of community awareness regarding disability^32^.
Technological						
Others			Long waiting period for hospital visits after referral^38^.	Lack of health insurance and inability to pay any service expenses^18^^,^^20^^,^^25^; and financial difficulties^14,16,34–36^.	Lack of involvement in making decisions about their health^28^.	Lack of request for and performance of preventive tests and care^20^ and lack of caregivers for adults with physical disabilities^39^.

LBI: *Lei Brasileira de Inclusão da Pessoa com Deficiência* (Brazilian Law for the Inclusion of Persons with Disabilities).

Considering the components of the theoretical framework of Levesque et al.^7^ (2013) and LBI^1^, Table 4 shows the barriers categorized according to the list of “who provides” (health professionals and services).

**Table 4:** Barrier categories according to components of the theoretical framework from Levesque et al.^7^ (2013) and LBI^1^ – health professionals and services.

Service provision: systems, institutions, organizations, and health providers						
Barriers	Approachability	Acceptability	Availability and accommodation	Affordability	Appropriateness	Others
Urbanistic			Poor access to buildings; difficulty in transporting wheelchairs^41^; physical barriers to care^16^; and accessibility difficulties^43^.		Distant health units^18^^,^^20^.	
Architectural			Poor parking facilities for disabled people, community and social environments generally unsuitable for wheelchair access^41^, and accessibility difficulties^43^.			
Transport			Lack of reliable and accessible transport for users to arrive at consultations^20^^,^^22^^,^^25^.			
Communication	Lack of communication on the part of professionals^31^^,^^44^; communication barriers between professionals and patients and between the health team itself^16^^,^^32^^,^^34^; language and cultural comprehension barriers^35^; and lack of information and educational interventions directed to users^43^.					
Attitudinal	Lack of education initiatives on health services and misunderstanding of rehabilitation by people with disabilities^32^.		Long waiting time for care^41^; failure to schedule consultations^32^; lack of personal motivation of professionals^32^; lack of patience of professionals; and lack of empathy and little understanding of disability issues^16^^,^^37^.		Lack of training and inadequate skills of professionals^46^; negative attitude and lack of coordinated care from professionals^18^^,^^20^; failures of health systems to respond adequately to the treatable morbidity identified^40^; lack of care for secondary health care^26^; lack of training and skills necessary for professionals and fragmentation of the health system^2^^,^^42^; lack of social support and discrimination^43^.	
Technological			Inadequate procedures for the treatment and maintenance of assistive products^41^.	Lack of funding for purchasing devices needed for treatment^44^; and barriers in the financing of rehabilitation^45^ and technologies^33^.	Barrier to the use of technology by therapists and lack of training and/or resources to support the use of technology in the practice^33^.	
Others			Fear and anxiety among professionals^36^.	Accessibility difficulties and financial constraints^43^.	Insufficient time for consultation^18^^,^^20^; difficulty in timely access to the appropriate services^27^; barriers to performing health tests^38^; discrimination from the health service provider^34^^,^^36^; lack of resources in the areas of psychology, speech therapy, and neuropsychology^32^; lack of policy-oriented self-care and health promotion^43^; limited availability of trained professionals and lack of support^43^.	

LBI: *Lei Brasileira de Inclusão da Pessoa com Deficiência* (Brazilian Law for the Inclusion of Persons with Disabilities).

## DISCUSSION

The disaggregation of access into broad dimensions, such as geographical, economic, or social aspects, allows more operational measures by studying specific determinants of access to health care. However, measuring access is a complex task when trying to include dimensions other than mere availability of services. Access is often perceived as being predominantly an attribute of services and is determined by factors such as availability, price and quality of health resources, goods, and services^7^.

Thus, the results of this study reveal that the population with disabilities faces several barriers to access health services. Problems in communication between professionals and patients and caregivers; financial issues; psychological, behavioral, and attitudinal issues; scarce provision of services; organizational and transport barriers stand out in general from the perspective of service users. These results are similar to those of other studies on the subject. Medeiros^47^ (2017), for example, showed that people with visual impairment experience various difficulties in accessing health services, including transport to visit the service, the physical access and the care itself, to communication with health professionals, violating the precepts of accessibility and interfering in the quality of health care for these people. Vieira et al.^48^ (2017) corroborated this fact, considering the perception of individuals with hearing impairment regarding health services. Barriers in communication between users and health professionals stood out, causing difficulties in access and doubts on the part of patients, as well as the absence of interpreters in the services. The presence of an assistant was frequently reported, questioning the implications of this fact for the bond between doctor and patient and for the privacy and autonomy of deaf individuals. The participants indicated dissatisfaction with the service.

We see health services accessibility as the result of the interaction of determinants of the characteristics of individuals (for example, the place where they live, their economic resources, and their social condition) and of services (for example, quantity, location of facilities, costs). The cost of the services itself is not the only determinant of accessibility of the services, but also the ability of people to pay for those services. Similarly, the location of a health facility will have an impact on access to health care, depending on the settlement patterns of the population it serves and its ability to reach the health service^7^.

França et al.^49^ (2016), report that aspects such as the precarious urban infrastructure of public services in the territories, local violence due to drug trafficking, lack of accessible public transportation, lack of professionals and facilities for continuity of care, are social determinants that make universal access to health by these groups even more complex and difficult, leading to hopelessness about the care provided by public services, disincentive in the search for care, lack of adherence and/or abandonment of treatments and care.

Corroborating the factors presented in another study, we noticed that, regarding transports to the health service, opinions diverge considerably, with variations in the time of travel, the need for an assistant, and the means of transport used. An important factor is that some feel the need to be accompanied and, in some cases, this can be explained by the lack of security in transiting the social environment alone, by the presence of possible architectural and social barriers or by their perspective of overprotection on the part of caregivers and family members^50^.

The research by Sousa et al.^50^ (2014) shows the obstacles faced by users of the system in the search for continuous and comprehensive care: restricted and non-welcoming access, excessive demand and absence of medical professionals in primary care, insufficient provision of consultations and specialized exams, long interval of time between specialized care, and lack of communication between services of different levels of care.

Among the results, the main barriers presented by service providers were: lack of training to professionals; failure of the health system; physical barriers; lack of resources/technology; and language barriers.

Using the literature review methodology, Amorim et al.^51^ (2018) highlight in their results the remarkably low qualification of primary health care professionals for the demands of users with some disability and emphasize that this requires regular opportunities for training. They also highlight that the lack of accessibility in health services was an obstacle to achieving comprehensive health, safeguarded by the Brazilian normative legal framework. Under this background, physical and attitudinal barriers present in most primary healthcare facilities contribute to an environment permeated by symbolic violence to people with some physical, intellectual, or sensory impairment. This fact perpetuates a cycle of inequities, in which people with disabilities are immersed, contributing to social exclusion.

By seeking to investigate knowledge of managers and health professionals about the main barriers in coverage and universal access to health by the extremely poor population, França et al.^49^ show that inadequate management and governance of local public policies, associated with the incoherent application of financial resources, lack of equipment, insufficient material and human resources to implement health and intersectoral problem-solving actions in primary health care, represent the main barriers in coverage and universal access to health by these individuals. Thus, financial barriers, including lack of resources, exist for all people who need access to the health service, including people with disabilities.

The lack of communication between the services and professionals that make up primary care and specialized care is another aspect that acts directly as an inhibitor of access and, consequently, points to the fragility in the constitution of the care network. Professionals hardly get together and communication, when it exists, is very incipient, by referral forms. The delay in scheduling specialized consultations is one of the barriers to population’s access to comprehensive care. What stands out more in the report from the professionals in this study is the anguish due to the consultation waiting time, which negatively impacts on the quality and problem-solving capacity of the care provided to the population^50^.

Other authors emphasize the need for investment in the training of professionals from the SUS service network since graduation and, even in service, for using LIBRAS (Brazilian Sign Language), in addition to expanding debates on communication, ethics, and citizenship, from the perspective of the social inclusion of these people with disabilities in all spheres of social life, as provided for in the legislation^51^.

In addition to the barriers, the articles identified by this review showed several equity-related aspects. Access to health services and equity are deeply related to the capacity of health systems to organize themselves to respond adequately to the needs of citizens^52^. When we analyzed the characteristics with potential to generate inequities in the 96 reviews included, issues related to the place of residence, race/ethnicity, gender/sex, education, socioeconomic status, social capital, factors that may lead to discrimination, factors related to caregivers such as family members and personal relationships stood out.

A study aiming to identify the main problems in access to health services for children with disabilities in Latin America points out that the difficulty of access is associated with demographic and socioeconomic factors, in addition to greater vulnerability. In addition, the following barriers stand out: mobility difficulties, family vulnerability, weak link between health services and the community, limited supply of specialized services, lack of health information, inefficient referrals, late diagnosis of comorbidities, and lack of public policies and of infrastructure^53^.

Efforts to mitigate barriers to equitable health services accessibility for people with disabilities are needed. Factors related to disparities in the access to health care among distinct socially vulnerable populations, such as underprivileged racial and ethnic groups, more vulnerable socioeconomic groups, and rural residents, additionally affect access for people with disabilities, who often experience poverty more than any minority or ethnic group. Many people with disabilities are caught in a cycle of poverty and deprivation, without the possibility of accessing education, work, and social facilities^41^. We also noted that simplifying the health information provided to patients as part of health services is needed^32^.

Considering the knowledge gaps that the articles present, more research is needed to understand how people with disabilities are accessing health services, not only in terms of use, but also in the coverage of preventive services, health services accessibility, and quality of received care^14^. Further studies aimed at developing effective training of the professional team are also needed, potentially based on the fundamental principles underlying the positive relationships between professional and patient identified by this review, such as effective communication, attitudes without judgment and encouraging active involvement in the treatment process^16^.

From a methodological point of view, additional high-quality research is needed to identify the characteristics of individuals with disabilities that face greater challenges in accessing health care^54,55^, in addition to better-designed and well-informed randomized clinical trials to build a stronger evidence base, recognizing methodological challenges, not only due to the complexity of the context and the variety of disabilities, but also to the additional challenges in conducting research in low- and middle-income countries. This would also allow grouping the results to conduct meta-analyses^56^.

Future studies should consider the role of technology in access, engagement, and navigation in the health system and the impact of intersectionality among marginalized groups^57^. A limitation of this scoping review on barriers is due to the process of searching and selecting studies. We must highlight the absence of qualitative research on the experiences of people with disabilities in accessing health services, not only regarding use, but also coverage of services and quality of care received. Another important point to consider is that most publications report data from studies conducted in high-income countries.

## CONCLUSIONS

With the scoping review process, we could observe that the lack of communication is a very important barrier and can occur in several contexts, between users and service providers, between service providers and health service management, and between health care levels. Thus, the failed communication between users and professionals hinders access to services at all levels and has the potential to negatively impact the autonomy of people with disabilities to their treatment. Economic, territory, and infrastructure aspects are barriers that greatly impact on users’ access to health services; and the lack of training of professionals, failure of the health system, physical barriers, lack of resources/technology, and language barriers are barriers that affect service providers.

All these barriers generate inequities, which result in increased social exclusion, so planning more appropriate strategies and actions also on the part of health services to enable them to respond to the needs of citizens is necessary.

All these factors indicate the need to include other search terms to cover these gaps or even expand to studies beyond literature reviews.

Another important point to consider is that most reviews report data from studies conducted in high-income countries.
