# Supplementary material for: Barriers to the access of people with disabilities to health services: a scoping review
Source: Rev Saude Publica. 2022 Jun 27;56:64. doi: 10.11606/s1518-8787.2022056003893 (PMC9239543; doi:10.11606/s1518-8787.2022056003893)
Supplement: Translation

# Translation: Barreiras ao acesso das pessoas com deficiência aos serviços de saúde: uma revisão de escopo

**Authors:** Karina Aparecida Padilha Clemente, Simone Vieira da Silva, Gislene Inoue Vieira, Maritsa Carla de Bortoli, Tereza Setsuko Toma, Vinícius Delgado Ramos, Christina May Moran de Brito

**Affiliations:** I Brasil Universidade de São Paulo. Faculdade de Medicina. Departamento de Medicina Legal, Ética Médica, Medicina Social e do Trabalho. São Paulo, SP, Brasil.; II Brasil Secretaria de Estado da Saúde de São Paulo. Instituto de Saúde. Departamento de Ciência e Tecnologia. São Paulo, SP, Brasil; III Brasil Hospital das Clínicas da Faculdade de Medicina da Universidade de São Paulo. Instituto de Medicina Física e Reabilitação. São Paulo, SP, Brasil; IV Brasil Hospital das Clínicas da Faculdade de Medicina da Universidade de São Paulo. Instituto do Câncer do Estado de São Paulo. São Paulo, SP, Brasil.

**Keywords:** Pessoas com Deficiência, Barreiras ao Acesso aos Cuidados de Saúde, Barreiras de Comunicação, Relações Médico-Paciente, Serviços de Saúde para Pessoas com Deficiência, Revisão

## Abstract

**OBJETIVO:**

Analisar as evidências científicas relativas às barreiras para o acesso de pessoas com deficiência aos serviços de saúde.

**MÉTODOS:**

Realizou-se uma revisão de escopo estabelecendo-se a perguntanorteadora: “Quais são as principais barreiras que as pessoas com deficiência enfrentam no acesso a serviços de saúde?” O levantamento dos artigos foi realizado em julho de 2019, em seis bases de dados de literatura científica. Dos 1.155 documentos identificados nas buscas, após seleção por título e resumo, foram lidas na íntegra 170 publicações e, após leitura, 96 artigos foram incluídos e categorizados conforme referencial teórico.

**RESULTADOS:**

As principais barreiras indicadas pelos usuários do serviço foram: comunicação falha entre profissionais e paciente/cuidador; limitações financeiras; questões atitudinais/comportamentais; oferta de serviços escassa; barreiras organizacionais e de transporte. As principais barreiras apresentadas pelos prestadores de serviços foram: falta de treinamento/capacitação aos profissionais; falha do sistema de saúde; barreiras físicas; falta de recursos/tecnologia e barreiras de idioma.

**CONCLUSÕES:**

Ficou evidente que as pessoas com deficiência enfrentam diversas barreiras ao tentarem acesso aos serviços de saúde de que necessitam e que usuários e profissionais de saúde têm visões distintas e complementares sobre as dificuldades.

## INTRODUÇÃO

A Lei Brasileira de Inclusão da Pessoa com Deficiência (LBI) destaca-se por incorporar ao ordenamento jurídico brasileiro um conceito de deficiência que a considera como o resultado da interação entre pessoas com impedimentos físicos, mentais, intelectuais ou sensoriais e barreiras ambientais incapacitantes^1^.

Segundo a Organização Mundial da Saúde (OMS) estima-se que haja um bilhão de pessoas com deficiência, constituindo 15% da população mundial^2^. No Brasil, o Censo Demográfico de 2010 estimou que 23,9% das pessoas tinham algum tipo de deficiência autorreferida e que 6,7% delas as consideravam deficiências “severas”^3^. Com o envelhecimento da população e com o aumento da prevalência das doenças crônicas não transmissíveis e incapacitantes, bem como comportamentos prejudiciais que podem causar danos à saúde, é provável que esses números aumentem^2^.

Essas pessoas podem ter necessidades de saúde específicas devido às suas deficiências e condições de saúde associadas. As evidências indicam que as pessoas com deficiência têm maiores necessidades de saúde do que as pessoas sem deficiência e que essas necessidades geralmente não são satisfeitas. Além das necessidades usuais de saúde, como a imunização, por exemplo, elas têm piores níveis de acesso aos serviços de saúde e piores desfechos de saúde, especialmente em países de baixo, médio/baixo e médio/alto níveis de renda, como o Brasil^2^. Essa situação é de fato evidente em nosso país, onde estudos indicam que pessoas com deficiências apresentam níveis mais altos de morbidade, comportamentos que causam prejuízos à saúde, e maior uso de serviços de saúde e hospitalizações^4^.

As pessoas com deficiência, portanto, necessitam de cuidados gerais adequados em saúde, que cubram todos os aspectos, incluindo os preventivos e promotores de saúde, amparados pela atenção primária, secundária e terciária, e também podem exigir cuidados de reabilitação e tratamento especializado – relacionados ou não à sua deficiência subjacente – para “otimizar a funcionalidade e reduzir a incapacidade”^2^.

Existem fortes evidências em todo o mundo de que as pessoas com deficiência têm dificuldades em acessar os serviços de saúde e isso também é observado nos poucos dados disponíveis nas pesquisas realizadas no Brasil. Por exemplo, a Pesquisa Nacional de Saúde brasileira mostrou baixo nível de acesso aos serviços de reabilitação entre essa população (5–30% em todos os tipos de deficiência), com alguma variação geográfica^5^. Apesar de as políticas e as leis brasileiras apoiarem fortemente a inclusão dessas pessoas no sistema de saúde, a epidemia de zika em 2015 revelou lacunas no Sistema Único de Saúde (SUS) em termos de atenção à saúde para pessoas com deficiência. Os cuidadores de crianças com síndrome congênita do vírus zika apresentam grandes dificuldades em acessar serviços relevantes, centrados nas pessoas, tanto em escala local quanto nacional^6^.

Ainda que fundamental para o desempenho dos sistemas de saúde em todo o mundo, o acesso aos cuidados de saúde, por pessoas com e sem deficiência, continua sendo um conceito complexo^7^. Os potenciais desafios enfrentados no acesso aos cuidados de saúde incluem discriminação, inacessibilidade física, inacessibilidade e indisponibilidade da informação. Em todo o mundo, faltam evidências sobre como promover a inclusão de pessoas com deficiência no sistema de saúde^2^.

Partindo do problema exposto, realizou-se uma revisão de escopo, cujo objetivo foi analisar as evidências científicas globais e locais sobre barreiras de acesso aos serviços de saúde para pessoas com deficiência.

## MÉTODOS

De acordo com o *Joanna Briggs Institute Reviewer’s Manual*^8^, uma revisão de escopo é composta pelas seguintes fases: 1) identificar a questão de pesquisa; 2) identificar estudos relevantes; 3) selecionar os estudos disponíveis; 4) mapear os dados; 5) coletar, resumir e relatar os resultados e 6) realizar o exercício de consulta, sendo que essa última fase é opcional e não foi aplicada neste trabalho. Esse tipo de síntese do conhecimento possui uma estrutura original e deve ser conduzida com rigor, transparência e confiabilidade. Ela tem sido utilizada por pesquisadores interessados em mapear a literatura científica e detectar lacunas do conhecimento^8^. Este manuscrito foi ancorado por uma versão específica do Prisma (*Preferred Reporting Items for Systematic reviews and Meta-Analyses extension for Scoping Reviews*, Prisma, 2020), uma lista de verificação com 22 itens elaborada para orientar o desenvolvimento de um relatório de revisão de escopo^9^.

A pergunta norteadora proposta para esta revisão foi: “Quais são as principais barreiras enfrentadas pelas pessoas com deficiência para acessar os serviços de saúde?”

Nesse estudo, para se explorar os achados a partir de uma perspectiva abrangente e dinâmica, adota-se a proposta de Levesque et al.^7^, que estruturam o conceito de acesso aos cuidados de saúde em múltiplos níveis e identificam os determinantes que podem afetá-lo a partir de fatores relacionados aos sistemas, instituições e profissionais de saúde, assim como aos indivíduos, às comunidades e à população.

Realizou-se uma busca sistematizada por evidências na literatura, publicadas em português, inglês ou espanhol, abordando os temas: pessoas com deficiência, acessibilidade aos serviços de saúde, serviços de saúde para pessoas com deficiência, disponibilidade de serviços de saúde, disparidades e iniquidades em saúde, acesso a cuidados de saúde, acesso aos serviços de saúde. Os descritores foram acompanhados dos operadores booleanos “*or*” e “*and*” na realização das buscas.

O levantamento bibliográfico foi realizado em julho de 2019, em seis bases de dados de literatura científica: PubMed, *Health Systems Evidence*, Scopus, Embase, *Health Evidence* e Lilacs conforme descrito no Quadro.

**Quadro:** Detalhamento do levantamento bibliográfico realizado nas bases de dados de literatura científica.

Base	Data	Estratégia
PubMed	06/07/2019	(((Disabled Person OR Person, Disabled OR Persons, Disabled OR Persons with Disabilities OR Disabilities, Persons with OR Disability, Persons with OR Persons with Disability OR Handicapped OR People with Disabilities OR Disabilities, People with OR People with Disability OR Physically Handicapped OR Handicapped, Physically OR Physically Disabled OR Disabled, Physically OR Physically Challenged)) AND (“Health Services Accessibility”[Mesh] OR Availability of Health Services OR Health Services Availability OR Accessibility, Health Services OR Access to Health Care OR Accessibility of Health Services OR Health Services Geographic Accessibility OR Program Accessibility OR Accessibility, Program)) AND (SYSTEMATIC REVIEW OR SYSTEMATIC REVIEWS)
PubMed	06/07/2019	(((Disabled Person OR Person, Disabled OR Persons, Disabled OR Persons with Disabilities OR Disabilities, Persons with OR Disability, Persons with OR Persons with Disability OR Handicapped OR People with Disabilities OR Disabilities, People with OR People with Disability OR Physically Handicapped OR Handicapped, Physically OR Physically Disabled OR Disabled, Physically OR Physically Challenged)) AND ((“Health Services for Persons with Disabilities”[Mesh] OR Health Services for the Disabled OR Health Services for People with Disabilities OR Health Services for Disabled Persons) AND Review[ptyp])) AND (SYSTEMATIC REVIEW OR SYSTEMATIC REVIEWS)
PubMed	06/07/2019	(((Disabled Person OR Person, Disabled OR Persons, Disabled OR Persons with Disabilities OR Disabilities, Persons with OR Disability, Persons with OR Persons with Disability OR Handicapped OR People with Disabilities OR Disabilities, People with OR People with Disability OR Physically Handicapped OR Handicapped, Physically OR Physically Disabled OR Disabled, Physically OR Physically Challenged)) AND ((“Healthcare Disparities”[Mesh] OR Disparity, Healthcare OR Health Care Inequalities OR Health Care Inequality OR Inequalities, Health Care OR Inequality, Health Care OR Healthcare Disparity OR Healthcare Inequalities OR Healthcare Inequality OR Inequalities, Healthcare OR Inequality, Healthcare OR Disparities, Healthcare OR Health Care Disparities OR Disparities, Health Care OR Disparity, Health Care OR Health Care Disparity) AND Review[ptyp])) AND (SYSTEMATIC REVIEW OR SYSTEMATIC REVIEWS)
*Health Systems Evidence*	06/07/2019	disability OR disabilities
*Health Evidence*	06/07/2019	[(disabled persons) OR disability OR disabilities]
Scopus	06/07/2019	TITLE-ABS-KEY(“disabled persons”ANDaccessibility)AND(LIMIT-TO(DOCTYPE,”re”))
EMBASE	06/07/2019	(‘disabled person’/exp OR ‘disabled person’ OR ‘disability’/exp OR disability) AND (‘accessibility’/exp OR accessibility) AND (‘health care facilities and services’/exp OR ‘health care facilities and services’) AND [review]/lim AND [embase]/lim
Lilacs via portal regional BVS	06/07/2019	(“pessoas com deficiência” OR “disabled persons” OR “personas con discapacidad” OR deficiência OR deficiências OR “deficiência física” OR “deficiências fisicas” OR “deficiente físico” OR incapacidade OR “incapacidade funcional” OR “limitação física” OR “pessoa com desvantagem” OR “pessoas com desvantagens” OR “pessoa com incapacidade” OR “pessoas com incapacidade” OR “pessoas com deficiências” OR “pessoas com incapacidades” OR “pessoa com incapacidade física” OR “pessoa com deficiência fisica” OR “pessoas com deficiência física” OR “pessoas com incapacidade física” OR “pessoas com deficiências físicas” OR “pessoa com limitação física” OR “pessoas com limitação física” OR “pessoas com limitações físicas” OR “pessoa com necessidade especial” OR “pessoas com necessidade especial” OR “pessoas com necessidades especiais”) AND (“acesso aos serviços de saúde” OR “health services accessibility” OR “accesibilidad a los servicios de salud” OR “disparidades em assistência à saúde” OR “healthcare disparities” OR “disparidades en atención de salud” OR “serviços de saúde para pessoas com deficiência” OR “health services for persons with disabilities” OR “servicios de salud para personas con discapacidad”) AND (instance:”regional”) AND ( db:(“LILACS” OR “BDENF” OR “BBO” OR “IBECS”))

O processo de seleção dos artigos por leitura de títulos, resumos e do texto completo, foi realizado em etapas subsequentes por uma dupla de revisores, de forma independente. No caso de discordâncias, a decisão foi tomada por um terceiro revisor.

O foco desta revisão esteve direcionado às revisões da literatura que abordassem deficiências física, auditiva, visual, intelectual e múltipla, não abrangendo as condições de saúde mental.

Artigos que abordavam uma condição ou necessidade de saúde específica ou comparavam diferentes formas de tratamentos, que não apresentaram questões relacionadas ao acesso das pessoas com deficiência ao sistema de saúde e publicações que não fossem apresentadas como revisão da literatura foram excluídas.

As principais informações das publicações foram sintetizadas em uma planilha criada especificamente para esta revisão, visando orientar as análises descritivas e críticas dos estudos selecionados. O processo de extração não foi realizado em duplicidade.

A planilha de extração continha os seguintes dados: base de dados da qual os artigos foram coletados; título; autor; ano de publicação; referência bibliográfica; desenho do estudo; quantidade de estudos e os desenhos que integravam cada documento; último ano de busca; objetivos; focos primário e secundário; tipos de deficiência (visual, auditiva, física, intelectual ou deficiências múltiplas); população do estudo e condição de saúde dos indivíduos que compunham as amostras; contexto dos elementos das intervenções analisadas; níveis dos serviços de saúde abrangidos (primário, secundário ou terciário); países onde os estudos foram realizados; estado ou região, em caso de artigos nacionais; informações sobre os sistemas de saúde contidas nos artigos; lacunas do conhecimento relevantes levantadas e observações sobre o estudo.

Também foram extraídos dos artigos informações sobre a quais componentes do sistema de saúde as barreiras estavam relacionadas, tendo como referencial teórico a publicação *“Strengthening Health Systems to Improve Health Outcomes – Everybody’s Business”* da Organização Mundial da Saúde (OMS)^10^, que considera os seguintes âmbitos: liderança e governança; força de trabalho; financiamento; tecnologias; sistemas de informação e modelos de atenção/prestação de serviços.

Informações sobre equidade foram coletadas, quando disponíveis, segundo o referencial teórico proposto pelo grupo de O’Neill et al.^11^, sintetizados no acrônimo Progress (*Place of Residence, Race, Occupation, Gender, Religion, Education, Socioeconomic status* and *Social capital*) acrescidos de fatores que podem originar discriminação, fatores relacionados aos cuidadores e a relações pessoais.

A categoria de extração central foi composta pelas barreiras e os facilitadores do acesso, identificados a partir das perspectivas das pessoas com deficiência, cuidadores, profissionais, serviços e o sistema de saúde.

As revisões incluídas não foram avaliadas quanto à qualidade metodológica, uma vez que a etapa é facultativa numa revisão de escopo.

Etimologicamente, o termo acesso apresenta noções complexas. O “acesso é uma dimensão do desempenho do sistema de saúde, associada à oferta”, enquanto a acessibilidade refere-se à “característica da oferta de serviço de saúde ou do ajuste entre a oferta e a população”, que podem ser medidos analisando os resultados por meio dos programas de avaliação do desempenho, no caso dos serviços de saúde^12^.

Quanto ao contexto da saúde, Levesque et al.^7^ propõem uma estrutura dinâmica composta por características dos provedores dos serviços de saúde e habilidades adquiridas pelos usuários desses serviços. As dimensões do acesso não são independentes, são inter-relacionadas, podendo influenciar umas às outras. Nesse contexto, o acesso é concebido em cinco dimensões, bem como cinco habilidades.

As cinco características manifestadas pelos sistemas, pelas instituições, pelas organizações e pelos provedores de saúde, bem como suas particularidades, são: *approachability* (abordagem), *acceptability* (aceitabilidade), *availability and accommodation* (disponibilidade e acomodação), *affordability* (acessibilidade financeira) e *appropriateness* (adequação).

As cinco habilidades atribuídas aos indivíduos, às famílias, às comunidades e às populações são: *ability to perceive* (habilidade de perceber), *ability to seek* (habilidade de procurar), *ability to reach* (habilidade de alcançar), *ability to pay* (habilidade de pagar) e *ability to engage* (habilidade de se envolver).

Fica evidente uma dinâmica articulada composta por ações práticas que interagem entre si. A depender da qualidade dessa interação, as dimensões do acesso e as habilidades podem constituir barreiras ou facilitadores.

Mais além, a Lei Federal 13.146/2015, a LBI^1^, também conhecida como Estatuto da Pessoa com Deficiência, em seu Art. 3º, apresenta especificamente seis tipos de barreiras que podem dificultar ou impossibilitar o acesso das pessoas com deficiência aos serviços de saúde. As barreiras são divididas em: urbanísticas, arquitetônicas, de transporte, de comunicação, atitudinais e tecnológicas.

Assim, a partir dos dados extraídos, analisou-se as barreiras categorizadas em termos de uso dos serviços de saúde (por usuários e cuidadores) e de oferta desses serviços (por profissionais, serviços e sistemas), que foram agrupados conforme as cinco dimensões do acesso^7^ e as seis possibilidades de barreiras apresentadas na LBI^1^.

## RESULTADOS

Dos 1.155 documentos identificados nas buscas, 234 publicações foram excluídas por duplicidade, totalizando 921 artigos para análise inicial. Após seleção por títulos e resumos, 170 artigos foram buscados na íntegra e, após a busca, 158 artigos foram avaliados para elegibilidade, dos quais 62 foram excluídos por não pertinência ao objetivo da pesquisa. Dessa forma, 96 artigos foram incluídos e tiveram seus dados extraídos e categorizados. Todo o processo de seleção e elegibilidade está representado na Figura.

**Figura:** Fluxograma do processo de seleção e elegibilidade de estudos, adaptado de Prisma^9^.

Os países de realização dos estudos incluídos foram classificados segundo critérios estabelecidos pelo código padrão de país ou área para uso estatístico da OMS, originalmente publicada como Série M, nº 49 e agora comumente referida como padrão M49^13^.

A maior parte dos artigos selecionados analisam dados de países na região das Américas (66,6%, n = 64), desses, 90% (n = 58) são da América do Norte. Na sequência, os países europeus são objeto de 55,2% (n = 53) das publicações, seguidos da Oceania (n = 36) e da Ásia (n = 33) com pouco mais de 30% cada uma e, por último, da África (6,2%, n = 6). Cabe ressaltar que alguns artigos incluem dados de mais de um país ou região e que 36,4% (n = 35) não especificam o local de sua realização.

Das 96 publicações incluídas, apenas quatro destacam pesquisas realizadas no Brasil^14^. Em relação aos tipos de deficiências encontradas, 21% das populações estudadas possuíam deficiência física; 17% deficiência intelectual; 8% visual; 7% auditiva; 2% deficiências múltiplas e 1% paralisia cerebral. Em 44% dos estudos, os autores não especificam os tipos de deficiência da população estudada.

Em relação ao nível de complexidade dos cuidados de saúde abrangidos pelos estudos, são variados os contextos de intervenção nos quais estão inseridos. Enquanto mais de um terço dos artigos não especifica o nível de atenção (n = 38), outros envolvem mais de um nível ou mesmo os três níveis. No nível da atenção básica há 26 trabalhos, seguidos de 30 pesquisas envolvendo a atenção especializada e 35 estudos pertinentes à atenção terciária.

Em relação aos anos em que os artigos foram publicados, o mais antigo foi publicado em 1997 (n = 1) e os mais recentes são de 2019 (n = 2). É notável um aumento no número de artigos publicados com o passar dos anos, sendo que em 2008 apenas quatro artigos foram encontrados, em 2011, nove foram achados, em 2014, encontrou-se 12, e em 2017, foram achados 15 artigos.

Na análise dos dados realizou-se uma divisão entre as barreiras identificadas pelos usuários e cuidadores e as barreiras identificadas pelos prestadores de serviços de saúde.

Em 10 revisões, as barreiras apresentadas pelos usuários, cuidadores e prestadores do serviço de saúde, de uma forma geral, são inespecíficas ao nível de atenção. Na oferta de serviços, 11 revisões são inespecíficas ao nível de atenção.

As barreiras encontradas nos estudos, sendo o uso ou oferta dos serviços, de acordo com os níveis de atenção são apresentadas nas Tabelas 1 e 2, demonstrando respectivamente a percepção dos usuários e dos prestadores de serviço. Considerando o uso do serviço, oito revisões relataram barreiras relacionadas ao nível de atenção básica, quatro à atenção especializada e duas ao nível terciário. As barreiras apresentadas pelos prestadores do serviço, referem-se a seis revisões sobre o nível de atenção básica, cinco de atenção especializada e três do nível terciário.

Na Tabela 1 é possível observar as barreiras ao acesso das pessoas com deficiências nos serviços de saúde, conforme nível de atenção, de acordo com a percepção dos usuários.

**Tabela 1:** Barreiras ao acesso das pessoas com deficiências nos serviços de saúde, conforme nível de atenção (usuários).

Nível de atenção	Barreiras encontradas na literatura
Básico	Acesso limitado a apoio psicológico e pouco ou nenhum apoio de psiquiatria baseada na comunidade^18^; dificuldade em encontrar médicos dispostos a cuidar de pacientes gravemente incapacitados e complexos^19^; dificuldade em encontrar serviços de saúde de que necessita^18^^,^^19^; falta da solicitação e realização de exames e cuidados preventivos^20^; falta de acesso a informações sobre saúde sexual reprodutiva, serviços de planejamento familiar e serviços pré-natais e pós-natais^21^; falta de apoio profissional^22^; falta de comunicação do profissional com o paciente ou cuidador^22–25^; falta de informações aos cuidadores sobre condição de saúde do paciente e acesso insuficiente aos serviços de atendimento infantil^25^; falta de seguro de saúde e incapacidade de pagar quaisquer despesas de serviços^18^^,^^20^^,^^25^; e medo e ansiedade em relação ao tratamento dos filhos com deficiência^20^.
Especializado	Falta de acesso aos serviços de saúde^26^; falta de comunicação e de acesso à informação^27–29^; falta de envolvimento na tomada de decisões sobre sua saúde^28^; e problema de localização das unidades de saúde e dificuldades de transporte para chegar até os serviços, bem como o custo do transporte^29^.
Alta complexidade	Barreiras organizacionais, sociais e físicas para acessar serviços de saúde^30^; falta de informações sobre sua saúde e sobre o tratamento^31^; e sensação de abandono por parte dos cuidadores em relação ao tratamento dos usuários^31^.
Nível de atenção não especificado	Dificuldades com transporte^32^^,^^33^; dificuldades financeiras^14,16,34–36^; discriminação de prestadores de serviços de saúde^14,16,34–36^; falta de apoio ao paciente no processo de reabilitação^32^; falta de comunicação por parte dos profissionais^37^^,^^38^; falta de conscientização da comunidade em relação à deficiência^32^; falta de cuidadores para adultos com deficiência física^39^; falta de informações “acessíveis” sobre o tratamento e os serviços de saúde^34^^,^^35^^,^^37^^,^^38^; e longo período de espera para consultas hospitalares após o encaminhamento^38^.

Na Tabela 2 é possível observar as barreiras ao acesso das pessoas com deficiências nos serviços de saúde, conforme nível de atenção, de acordo com a oferta dos serviços.

**Tabela 2:** Barreiras ao acesso das pessoas com deficiências nos serviços de saúde, conforme nível de atenção (oferta do serviço).

Nível de atenção	Barreiras encontradas na literatura
Básico	Falta de transporte confiável e acessível para os usuários chegarem nas consultas^20^^,^^22^^,^^25^; falta de treinamento e habilidades inadequadas por parte de profissionais, questões ambientais como unidades de saúde distantes, tempo insuficiente para as consultas, atitude negativa e falta de cuidados coordenados por parte dos profissionais^18^^,^^20^; e falhas dos sistemas de saúde em responder adequadamente à morbidade tratável identificada^40^.
Especializado	Acesso deficiente às edificações, dificuldade de transportar cadeiras de rodas e instalações precárias de estacionamento para deficientes físicos, ambientes comunitários e sociais geralmente impróprios para o acesso de cadeira de rodas^41^; dificuldade no acesso oportuno a serviços apropriados^27^; falta de atenção a cuidados secundários em saúde^26^; falta de treinamento e habilidades necessárias para os profissionais e fragmentação do sistema de saúde^28^^,^^42^; e longo tempo de espera para atendimento e procedimentos inadequados para os tratamentos e manutenção de produtos assistivos^41^.
Alta complexidade	Ausência de autocuidado orientado a políticas e promoção de saúde, dificuldades de acessibilidade, disponibilidade limitada de profissionais treinados, falta de apoio social, discriminação, restrições financeiras, falta de informação e intervenções educacionais direcionadas aos usuários^43^; falta de comunicação por parte dos profissionais^31^^,^^44^ e falta de financiamento para compra de aparelhos necessários para tratamento^44^.
Nível de atenção não especificado	Barreira ao uso da tecnologia por terapeutas, falta de treinamento e/ou recursos para apoiar o uso de tecnologia na prática^33^, barreiras de comunicação entre profissionais e pacientes e entre a própria equipe de saúde^16^^,^^32^^,^^34^; barreiras de idioma e de compreensão cultural^35^; barreiras físicas ao cuidado^16^; barreiras no financiamento da reabilitação^45^ e de tecnologias^33^; barreiras para a realização de exames de saúde^38^; discriminação do prestador de serviços de saúde^34^^,^^36^; falha no agendamento de consultas^32^; falta de habilidades dos profissionais^46^; falta de iniciativas de educação sobre serviços de saúde e incompreensão da reabilitação pelas pessoas com deficiências^32^; falta de motivação pessoal dos profissionais^32^; falta de paciência dos profissionais, falta de empatia, pouca compreensão das questões relativas à deficiência^16^^,^^37^; falta de recursos nas áreas de psicologia, fonoaudiologia e neuropsicologia^32^; e medo e ansiedade entre os profissionais^36^.

Levando em consideração os componentes do referencial teórico Levesque et al.^7^ (2013) e a LBI^1^, as barreiras foram categorizadas de acordo a relação de “quem recebe”, usuários e cuidadores, conforme Tabela 3.

**Tabela 3:** Apresentação das categorias de barreiras conforme componentes do referencial teórico, Levesque et al.^7^ (2013) e LBI^1^ – usuários e cuidadores.

Uso do serviço: indivíduos, famílias, comunidades e populações						
Barreiras	Habilidade de perceber	Habilidade de procurar	Habilidade de alcançar	Habilidade de pagar	Habilidade de se envolver	Outras
Urbanísticas			Problema de localização das unidades de saúde^29^.			
Arquitetônicas			Barreiras organizacionais, sociais e físicas para acessar serviços de saúde^30^.			
Transporte			Dificuldades de transporte^29^^,^^32^^,^^33^ para chegar até as unidades de saúde, bem como o custo do transporte^29^.			
Comunicação			Falta de acesso a informações sobre sua saúde^27–29^, sobre o tratamento e os serviços de saúde^31,34–37^, e sobre saúde sexual reprodutiva^21^; e falta de comunicação^37^ do profissional com o paciente ou cuidador^22^^,^^25^^,^^37^^,^^38^.			
Atitudinais	Falta de acesso a serviços de planejamento familiar e serviços pré-natais e pós-natais^21^; acesso insuficiente aos serviços de atendimento infantil^25^; falta de acesso aos serviços de saúde^28^; sensação de abandono por parte dos cuidadores em relação ao tratamento dos usuários^31^; discriminação de prestadores de serviços de saúde^14,16, 34–36^; e falta de apoio ao paciente no processo de reabilitação^32^.	Medo e ansiedade em relação ao tratamento dos filhos com deficiência^20^; falta de apoio profissional^22^; acesso limitado ao apoio psicológico e pouco ou nenhum apoio de psiquiatria baseada na comunidade^18^; dificuldade em encontrar médicos dispostos a cuidar de pacientes gravemente incapacitados e complexos^19^; e dificuldade em encontrar serviços de saúde de que necessita^18^^,^^19^.				Falta de conscientização da comunidade em relação à deficiência^32^.
Tecnológicas						
Outras			Longo período de espera para consultas hospitalares após o encaminhamento^38^.	Falta de seguro de saúde e incapacidade de pagar quaisquer despesas de serviços^18^^,^^20^^,^^25^; e dificuldades financeiras^14,16,34–36^.	Falta de envolvimento na tomada de decisões sobre sua saúde^28^.	Falta da solicitação e realização de exames e cuidados preventivos^20^ e falta de cuidadores para adultos com deficiência física^39^.

LBI: Lei Brasileira de Inclusão da Pessoa com Deficiência

Levando em consideração componentes do referencial teórico, Levesque et al.^7^ (2013) e a LBI^1^, as barreiras foram categorizadas de acordo a relação de “quem oferece”, profissionais e serviços de saúde, conforme Tabela 4.

**Tabela 4:** Apresentação das categorias de barreiras conforme componentes do referencial teórico Levesque et al.^7^ (2013) e LBI^1^ – profissionais e serviços de saúde.

Oferta do serviço: sistemas, instituições, organizações e provedores de saúde						
BARREIRAS	Abordagem	Aceitabilidade	Disponibilidade e acomodação	Acessibilidade financeira	Adequação	Outras
Urbanísticas			Acesso deficiente às edificações; dificuldade de transportar cadeiras de rodas^41^; barreiras físicas ao cuidado^16^; e dificuldades de acessibilidade^43^.		Unidades de saúde distantes^18^^,^^20^.	
Arquitetônicas			Instalações precárias de estacionamento para deficientes físicos, ambientes comunitários e sociais geralmente impróprios para o acesso de cadeira de rodas^41^ e dificuldades de acessibilidade^43^.			
Transporte			Falta de transporte confiável e acessível para os usuários chegarem nas consultas^20^^,^^22^^,^^25^.			
Comunicação	Falta de comunicação por parte dos profissionais^31^^,^^44^; barreiras de comunicação entre profissionais e pacientes e entre a própria equipe de saúde^16^^,^^32^^,^^34^; barreiras de idioma e de compreensão cultural^35^; e falta de informação e intervenções educacionais direcionadas aos usuários^43^.					
Atitudinais	Falta de iniciativas de educação sobre serviços de saúde e incompreensão da reabilitação pelas pessoas com deficiências^32^.		Longo tempo de espera para atendimento^41^; falha no agendamento de consultas^32^; falta de motivação pessoal dos profissionais^32^; falta de paciência dos profissionais; e falta de empatia e pouca compreensão das questões relativas à deficiência^16^^,^^37^.		Falta de treinamento e habilidades inadequadas por parte de profissionais^46^; atitude negativa e falta de cuidados coordenados por parte dos profissionais^18^^,^^20^; falhas dos sistemas de saúde em responder adequadamente à morbidade tratável identificada^40^; falta de atenção a cuidados secundários em saúde^26^; falta de treinamento e habilidades necessárias para os profissionais e fragmentação do sistema de saúde^2^^,^^42^; falta de apoio social e discriminação^43^.	
Tecnológicas			Procedimentos inadequados para os tratamentos e manutenção de produtos assistivos^41^.	Falta de financiamento para compra de aparelhos necessários para tratamento^44^; e barreiras no financiamento da reabilitação^45^ e de tecnologias^33^.	Barreira ao uso da tecnologia por terapeutas e falta de treinamento e/ou recursos para apoiar o uso de tecnologia na prática^33^.	
Outras			Medo e ansiedade entre os profissionais^36^.	Dificuldades de acessibilidade e restrições financeiras^43^.	Tempo insuficiente para a consulta^18^^,^^20^; dificuldade no acesso oportuno a serviços apropriados^27^; barreiras para a realização de exames de saúde^38^; discriminação do prestador de serviços de saúde^34^^,^^36^; falta de recursos nas áreas de psicologia, fonoaudiologia e neuropsicologia^32^; ausência de autocuidado orientado a políticas e promoção de saúde^43^; disponibilidade limitada de profissionais treinados e falta de apoio^43^.	

LBI: Lei Brasileira de Inclusão da Pessoa com Deficiência

## DISCUSSÃO

A desagregação do acesso em amplas dimensões, como aspectos geográficos, econômicos ou sociais, permite medidas mais operacionais por meio do estudo dos determinantes específicos do acesso aos cuidados de saúde. No entanto, medir o acesso é uma tarefa complexa ao tentar incluir outras dimensões além da mera disponibilidade de serviços. O acesso é frequentemente percebido como sendo predominantemente um atributo de serviços e é determinado por fatores como a disponibilidade, preço e qualidade dos recursos, bens e serviços de saúde^7^.

Sendo assim, os resultados deste estudo revelam que a população com deficiência enfrenta diversas barreiras para o acesso aos serviços de saúde. Podem ser destacadas, de uma forma geral, na perspectiva dos usuários do serviço, problemas na comunicação entre profissionais e pacientes e cuidadores; questões financeiras; questões psicológicas, comportamentais e atitudinais; escassez na oferta de serviços; barreiras organizacionais e de transporte. Esses resultados são similares aos de outras pesquisas realizadas sobre o tema. Medeiros^47^ (2017), por exemplo, evidenciou que as pessoas com deficiência visual vivenciam diversas dificuldades no acesso aos serviços de saúde, que vão desde o deslocamento ao serviço, passando pelo acesso físico e atendimento, até a comunicação com os profissionais de saúde, infringindo os preceitos da acessibilidade e interferindo na qualidade da assistência à saúde dessas pessoas. Tal fato foi corroborado por Vieira et al.^48^ (2017), ao levar em conta a percepção de sujeitos com deficiência auditiva em relação ao seu atendimento nos serviços de saúde. Barreiras na comunicação entre os usuários e os profissionais de saúde foram ressaltadas, provocando dificuldades no acesso e dúvidas por parte dos pacientes. A ausência de intérpretes nos serviços foi evidenciada. A presença do acompanhante foi relatada frequentemente, questionando-se as implicações desse fato para o vínculo entre médico e paciente e para a privacidade e autonomia dos sujeitos surdos. Os participantes indicaram insatisfação com o atendimento.

Vemos o acesso aos serviços de saúde como o resultado da interação de determinantes das características dos indivíduos (por exemplo, o lugar onde vivem, seus recursos econômicos e sua condição social) e dos serviços (por exemplo, quantidade, localização das instalações, custos). Não é apenas o custo dos serviços em si que determina se os serviços são acessíveis, mas também a capacidade das pessoas de pagar por esses serviços. Da mesma forma, a localização de uma unidade de saúde terá impacto sobre o acesso aos cuidados de saúde, dependendo dos padrões de assentamento da população que atende e de sua capacidade de locomoção até o serviço de saúde^7^.

França et al.^49^ (2016), relatam que aspectos como a precária infraestrutura urbana dos serviços públicos nos territórios, violência local pelo narcotráfico, ausência de transporte público acessível, falta de profissionais e equipamentos para continuidade dos cuidados, são determinantes sociais que tornam o acesso universal à saúde por esses grupos ainda mais complexo e difícil, ocasionando-lhes desesperança quanto aos atendimentos ofertados pelos serviços públicos, desestímulo na busca por assistência, falta de adesão e/ou o abandono dos tratamentos e cuidados instituídos.

Corroborando os fatores apresentados em outro estudo, pôde-se perceber que, quando se trata do deslocamento ao serviço de saúde, há considerável diversidade de opiniões, com variações quanto ao tempo de deslocamento, à necessidade de acompanhante e ao meio de transporte utilizado. Um fator importante é que alguns sentem a necessidade de serem acompanhados e, em alguns casos, isso pode ser explicado pela falta de segurança em transitar no meio social sozinho, pela presença de possíveis barreiras arquitetônicas e sociais ou pelo sentimento de superproteção por parte dos cuidadores e familiares^50^.

Na pesquisa de Sousa et al.^50^ (2014), os obstáculos enfrentados pelos usuários do sistema na busca pelo cuidado contínuo e integral são apresentados: acesso restrito e sem acolhimento, excesso de demanda e ausência de profissionais médicos na atenção básica, insuficiência na oferta de consultas e exames especializados, longo intervalo de tempo entre os atendimentos especializados e ausência de comunicação entre os serviços de diferentes níveis assistenciais.

Dentre os resultados apresentados pelos prestadores de serviços, destacam-se as seguintes barreiras: falta de treinamento/ capacitação aos profissionais; falha do sistema de saúde; barreiras físicas; falta de recursos/tecnologia e barreiras de idioma.

Utilizando-se da metodologia de revisão da literatura, Amorim et al.^51^ (2018) destacam em seus resultados que é notável a baixa qualificação dos profissionais da atenção primária à saúde para as demandas dos usuários com alguma deficiência e salientam que isso requer espaços regulares de capacitação. Destacam também, que a falta de acessibilidade nos serviços de saúde mostrou-se um obstáculo ao alcance da integralidade em saúde, salvaguardada pelo arcabouço jurídico normativo brasileiro. Sob esse pano de fundo, ainda são presentes barreiras físicas e atitudinais na maioria das unidades básicas de saúde, contribuindo para uma ambiência permeada por violência simbólica às pessoas com alguma limitação ou impedimento físico, intelectual ou sensorial. Esse fato perpetua um ciclo de iniquidades, no qual as pessoas com deficiência estão imersas, contribuindo para a exclusão social.

Ao buscar investigar conhecimentos de gestores e profissionais da saúde sobre as principais barreiras na cobertura e acesso universal à saúde pela população extremamente pobre, França et al.^49^ mostra que a inadequada gestão e governança das políticas públicas locais, associadas à incoerente aplicação dos recursos financeiros, falta de equipamentos, recursos materiais e humanos insuficientes, para implantar ações de saúde e intersetoriais resolutivas na atenção primária à saúde, representam as principais barreiras na cobertura e no acesso universal à saúde por esses sujeitos. Dessa forma, as barreiras financeiras, incluindo a falta de recursos, está presente para todas as pessoas que precisam acessar o serviço de saúde, inclusive às pessoas com deficiência.

A ausência de comunicação entre os serviços e profissionais que compõem a atenção básica e a atenção especializada é também um aspecto que atua diretamente como inibidor do acesso e, consequentemente, aponta para a fragilidade na constituição da rede assistencial. Os profissionais dificilmente se reúnem e a comunicação, quando existe, acontece de forma muito incipiente, por meio das fichas de referência e contrarreferência. A demora no agendamento das consultas especializadas é uma das barreiras de acesso para o atendimento integral da população. O que chama mais atenção no relato dos profissionais neste estudo, é a angústia pelo tempo de espera das consultas, que impacta negativamente na qualidade e resolutividade do atendimento prestado à população^50^.

Outros autores ressaltam a necessidade de investimento na capacitação e formação de profissionais da rede de serviços do SUS desde a graduação e, mesmo em serviço, para o uso da Língua Brasileira de Sinais (Libras), além da ampliação de debates sobre comunicação, ética e cidadania, na perspectiva da inclusão social dessas pessoas com deficiência em todas as esferas da vida social, como previsto na legislação^51^.

Além das barreiras, diversos aspectos relacionados à equidade foram encontrados nos artigos identificados por essa revisão. O acesso a serviços de saúde e a equidade estão profundamente relacionados à capacidade dos sistemas de saúde de se organizarem visando uma resposta adequada às necessidades dos cidadãos^52^. Quando foram analisadas as características com potencial de gerar iniquidades nas 96 revisões incluídas destacaram-se questões relacionadas ao local de residência, raça/etnia, gênero/sexo, escolaridade, estado socioeconômico, capital social, fatores que podem originar discriminação, fatores relacionados aos cuidadores como familiares e relações pessoais.

Um estudo que objetivou identificar os principais problemas no acesso a serviços de saúde para crianças com deficiência na América Latina ressalta que a dificuldade de acesso está associada a fatores demográficos e socioeconômicos, além de maior vulnerabilidade. Além disso, destacam-se as seguintes barreiras: dificuldades de mobilidade, vulnerabilidade familiar, baixo vínculo dos serviços de saúde com a comunidade, baixa oferta de serviços especializados, falta de informações em saúde, encaminhamentos ineficientes, diagnóstico tardio de comorbidades e falta de políticas públicas e de infraestrutura^53^.

São necessários esforços visando mitigar as barreiras para que o acesso aos serviços de saúde seja equitativo para as pessoas com deficiência. Fatores relacionados às disparidades no acesso aos cuidados em saúde entre populações vulneráveis socialmente distintas, como grupos raciais e étnicos carentes, grupos socioeconômicos mais vulneráveis e residentes rurais, também afetam adicionalmente o acesso de pessoas com deficiência, que frequentemente experimentam a pobreza mais do que qualquer minoria ou grupo étnico. Muitas pessoas com deficiência são apanhadas em um ciclo de pobreza e privação, sem a possibilidade de acessar a educação, o trabalho e as instalações sociais^41^. Foi também observado que há necessidade de simplificar as informações de saúde fornecidas aos pacientes como parte dos serviços de saúde^32^.

Levando em consideração as lacunas de conhecimento que os artigos apresentam, são necessárias mais pesquisas para entender como as pessoas com deficiência estão acessando os serviços de saúde, não apenas em termos de utilização, mas também na cobertura de serviços preventivos, acessibilidade dos serviços de saúde e qualidade dos cuidados recebidos^14^. Também são necessários mais estudos que visem o desenvolvimento de um treinamento eficaz da equipe profissional, potencialmente baseando-se nos princípios fundamentais subjacentes às relações positivas entre profissional e paciente identificadas por esta revisão, como comunicação eficaz, atitudes sem julgamento e encorajando o envolvimento ativo no processo de tratamento^16^.

Do ponto de vista metodológico, pesquisas adicionais de alta qualidade são necessárias para identificar as características dos indivíduos com deficiência que enfrentam maiores desafios no acesso aos cuidados em saúde^54,55^, além de ensaios clínicos randomizados mais bem elaborados e bem informados para construir uma base mais sólida de evidências, reconhecendo os desafios metodológicos, não apenas devidos à complexidade do contexto e à variedade de deficiências, mas também aos desafios adicionais na realização de pesquisas em países de baixa e média renda. Isso também permitiria agrupar os resultados e realizar metanálises^56^.

Estudos futuros devem considerar o papel da tecnologia no acesso, engajamento e navegação no sistema de saúde e o impacto da interseccionalidade entre grupos marginalizados^57^. Uma limitação desta revisão de escopo sobre barreiras é decorrente do processo de busca e seleção dos estudos. A ausência de pesquisas qualitativas sobre experiências de pessoas com deficiência no acesso aos serviços de saúde, não apenas em termos de utilização, mas também quanto à cobertura de serviços e qualidade dos cuidados recebidos merece ser destacada. Outro ponto importante a se considerar é que a maioria das publicações relata dados de estudos realizados em países de alta renda.

## CONCLUSÕES

Por meio do processo de revisão de escopo, foi possível observar que a falta de comunicação é uma barreira muito importante e pode ocorrer em diversos contextos, entre usuários e prestadores de serviço, entre prestadores de serviço e gestão dos serviços de saúde e entre os níveis de atenção à saúde. Dessa forma, a comunicação falha entre os usuários e os profissionais não obstaculariza apenas o acesso aos serviços em todos os níveis, mas tem potencial para impactar negativamente a autonomia da pessoa com deficiência ao seu tratamento. Aspectos econômicos, de território e de infraestrutura são barreiras que apresentam grande impacto para o acesso dos usuários aos serviços de saúde, assim como a falta de treinamento e capacitação dos profissionais; falha do sistema de saúde; barreiras físicas; falta de recursos/tecnologia e barreiras de idioma são barreiras encontradas que afetam os prestadores de serviço.

Todas essas barreiras geram iniquidades, o que acaba resultando em aumento da exclusão social, por isso, faz-se necessário o planejamento de estratégias e ações mais adequadas por parte, também, dos serviços de saúde, para que possam ser capazes de responder às necessidades dos cidadãos.

Todos esses fatores indicam a necessidade de incluir outros termos de busca para cobrir essas lacunas ou mesmo ampliar para estudos além das revisões de literatura.

Outro ponto importante a se considerar é que a maioria das revisões relata dados de estudos realizados em países de alta renda.
